# Including all voices in international data-sharing governance

**DOI:** 10.1186/s40246-018-0143-9

**Published:** 2018-03-07

**Authors:** Jane Kaye, Sharon F. Terry, Eric Juengst, Sarah Coy, Jennifer R. Harris, Don Chalmers, Edward S. Dove, Isabelle Budin-Ljøsne, Clement Adebamowo, Emilomo Ogbe, Louise Bezuidenhout, Michael Morrison, Joel T. Minion, Madeleine J. Murtagh, Jusaku Minari, Harriet Teare, Rosario Isasi, Kazuto Kato, Emmanuelle Rial-Sebbag, Patricia Marshall, Barbara Koenig, Anne Cambon-Thomsen

**Affiliations:** 1 Centre for Health Law and Emerging Technologies, NDPH, University of Oxford, Ewert House, Ewert Place, Summertown, Oxford, OX2 7DD UK; 2 Melbourne Law School, University of Melbourne, 185 Pelham Street, Carlton, Victoria 3053 Australia; 3 Genetic Alliance USA, 4301 Connecticut Ave NW, Suite 404, Washington DC, 20008-2369 USA; 4 Center for Bioethics, University of North Carolina at Chapel Hill, 333 McNider Hall, Chapel Hill, NC 27599-7240 USA; 5 Department of Genetics and Bioinformatics, Norwegian Institute of Public Health, PO Box 4404, Nydalen, 0403 Oslo, Norway; 6 Faculty of Law, University of Tasmania, Private Bag 89, Hobart, Tasmania 7001 Australia; 7 School of Law, University of Edinburgh, Old College, South Bridge, Edinburgh, EH8 9YL UK; 8 Cohort Studies, Norwegian Institute of Public Health, PO Box 4404, Nydalen, 0403 Oslo, Norway; 9 Center for Bioethics and Research, Ibadan, Nigeria; 10 Institute of Human Virology Nigeria, Abuja, Nigeria; 11 Greenebaum Comprehensive Cancer Center and Institute of Human Virology, University of Maryland School of Medicine, 725 W. Lombard St. Suite 445, Baltimore, MD 21201 USA; 12 International Centre for Reproductive Health, University of Gent, De Pintepark II, De Pintelaan 185, 9000 Ghent, Belgium; 13 Institute for Science, Innovation and Society, University of Oxford, 64 Banbury Road, Oxford, OX2 6PN UK; 14 Policy, Ethics and Life Sciences (PEALS) Research Centre, Newcastle University, Newcastle upon Tyne, NE1 7RU UK; 15 Uehiro Research Division for iPS Cell Ethics, Center for iPS Cell Research and Application, Kyoto University, 53 Kawahara-cho, Shogoin, Sakyo-ku, Kyoto, 606-8507 Japan; 16 Institute for Bioethics and Health Policy, Department of Human Genetics, Leonard M. Miller School of Medicine, University of Miami, 1501 NW 10th Avenue, Biomedical Research Building (BRB) Room 361, Miami, FL 33136 USA; 17 Department of Biomedical Ethics and Public Policy, Graduate School of Medicine, Osaka University, 2-2 Yamadaoka, Suita, Osaka, 565-0871 Japan; 18 National Institute for Research and Health (Inserm), UMR 1027 Inserm, Toulouse University, 37 allées Jules Guesde, 31000 Toulouse, France; 19 Department of Bioethics, School of Medicine, TA200, Case Western Reserve University, 10900 Euclid Avenue, Cleveland, OH 44106-4976 USA; 20 UCSF School of Nursing, Institute for Health and Aging, University of California, San Francisco, 3333 Calif. St, Laurel Heights, San Francisco, CA 94118 USA; 21 CNRS, Toulouse, France; Joint research unit on epidemiology and public health, Inserm (National Institute for Health and Medical Research) and University Toulouse III Paul Sabatier, Toulouse, France

**Keywords:** Data sharing, International research, Governance, Public engagement, Inclusion, Digital technologies

## Abstract

**Background:**

Governments, funding bodies, institutions, and publishers have developed a number of strategies to encourage researchers to facilitate access to datasets. The rationale behind this approach is that this will bring a number of benefits and enable advances in healthcare and medicine by allowing the maximum returns from the investment in research, as well as reducing waste and promoting transparency. As this approach gains momentum, these data-sharing practices have implications for many kinds of research as they become standard practice across the world.

**Main text:**

The governance frameworks that have been developed to support biomedical research are not well equipped to deal with the complexities of international data sharing. This system is nationally based and is dependent upon expert committees for oversight and compliance, which has often led to piece-meal decision-making. This system tends to perpetuate inequalities by obscuring the contributions and the important role of different data providers along the data stream, whether they be low- or middle-income country researchers, patients, research participants, groups, or communities. As research and data-sharing activities are largely publicly funded, there is a strong moral argument for including the people who provide the data in decision-making and to develop governance systems for their continued participation.

**Conclusions:**

We recommend that governance of science becomes more transparent, representative, and responsive to the voices of many constituencies by conducting public consultations about data-sharing addressing issues of access and use; including all data providers in decision-making about the use and sharing of data along the whole of the data stream; and using digital technologies to encourage accessibility, transparency, and accountability. We anticipate that this approach could enhance the legitimacy of the research process, generate insights that may otherwise be overlooked or ignored, and help to bring valuable perspectives into the decision-making around international data sharing.

Modern biomedical research has been accelerated by advances in digital technologies [1], enabling interdisciplinary collaborations to collect, analyze and share different kinds of data across the globe. To facilitate data sharing, governments, funding bodies, institutions, and publishers have developed data infrastructures, policies, and incentives. Global data sharing is anticipated by many commentators to boost progress in health and medicine, reduce research waste, increase research value, and promote research transparency [2]. While most research data are generated by researchers, digital innovation has enabled patients and citizens to be involved in data collection and creation through the use of smart phones and devices, and involvement in citizen-science projects. The scope of data considered to be health-related has also expanded to include demographic, administrative, socio-economic, medical, and behavioral measures as well as genomics and multi-omics data which can be derived from a number of different sources.

While data sharing is strongly encouraged, concerns exist that the existing governance frameworks of data sharing are inappropriate to cope with the size, scope, complexity, and scale of data sharing that is required in modern health research [3–5]. Furthermore, we argue that these frameworks tend to perpetuate, or even exacerbate, many of the current inequalities in research and health by obscuring the important role of those who donate data, whether they be low- or middle-income country researchers, patients, research participants, groups, or communities [6]. This paper will discuss some of the inadequacies of the existing governance systems for research and suggest an approach to bring the voices of data contributors into decision-making around data sharing.

## Flaws of the current data-sharing governance frameworks

Current data-sharing governance frameworks are not attuned to the needs of multi-institutional or multi-jurisdictional health research [3]. In most countries, governance systems primarily are tied to territorial jurisdictions; these systems were originally developed to protect personal data within a single jurisdiction. Efforts to combine or sequentially apply territorial governance structures across borders may lead to undue complexity and bureaucratic regulatory thickets resulting in inefficient data flows characterized by delays in the research progress [7, 8]. Applying local or territorial governance to broader areas overloads the structures to a point where they are either inappropriate or lose their moral legitimacy by failing to reflect the concerns, interests, and values of all of the data contributors involved. This means that the movement of data becomes a series of contractual transactions or transfers in which it is very difficult to know if data have been used according to the wishes of data donors and curators further upstream in the research process as the data move through global networks.

Such a system also has the tendency to perpetuate inequalities by obscuring the contributions of different stakeholders along the data stream. This is particularly problematic if it creates a proportionately greater disadvantage for people in resource-poor countries to contribute to, be recognized for, and benefit from science. It also does not recognize the connectivity between data providers [9], and may contribute to muting the voices of the data contributors as data move progressively away from them. This lack of connection between the data providers and those who use the data can lead to potentially unjust outcomes that are not in accordance with the expectations of those who are providing data. Ultimately most data on individuals requires consent to be obtained. If data are shared in ways that appear contrary to the interests of the subjects and donors of that data, this could lead to a loss of trust in the research process and to people withholding consent and refusing to participate in further research.

Institutional Review Boards [10] or Research Ethics Committees [7] are the key governance bodies for research overseeing the recruitment of participants into the initial study, when data are generated. These committees often are based locally and must make decisions on behalf of participants domiciled within their jurisdictions. In multi-institutional research, several such committees perform their reviews in a succeeding manner with the same goal of protecting the interests of local participants, researchers, and institutions. This can lead to differences in decision-making, not only just between national committees, but also committees within the same jurisdiction. Although there may be patients and participants sitting on these committees as representatives, decisions are made on behalf of research participants by experts. The end result of this piece-meal decision-making is that it may not be in accordance with what individuals, groups, and communities want in terms of international data sharing [11]. While in many regions of the world such as sub-Saharan Africa, mechanisms have been developed to engage communities and obtain their consent before research implementation [12, 13], such mechanisms are not broadly adopted in western countries or other countries with advanced biomedical research. Generally, community perspectives and formal public consultations are not requirements in the deliberations and decision-making regarding international data sharing.

## Proposed new data-sharing governance frameworks

Recently, platforms have been developed to support the use of scientific data and to address some of the challenges described above. Examples include the newly proposed European Open Science Cloud [14], the Global Alliance for Genomics and Health [15, 16], the Database of Genotypes and Phenotypes (dbGaP) [17], and the European Genome-phenome Archive [18]. Within these managed access platforms, new forms of governance are applied that sit alongside the traditional oversight bodies of expert ethics committees. For instance, Data Access Committees have become a standard oversight body for the approval of access to research platform by researchers [19], with some committees undertaking responsibility for a number of studies such as in the case of METADAC [20]. To facilitate access to data, new governance mechanisms such as ‘pop-up’ governance [21] have been developed by research consortia. In such projects, data access committees are established at a consortium level and provide greater control for the researchers who have collected the data, and, potentially, for the participants who donate their data as well.

Many of these initiatives have largely been instigated by researchers and funding bodies in high-resourced countries, supported by foundational documents such as the Fort Lauderdale Agreement of 2003 [22] and the Toronto Statement of 2009 [23]. These governance systems have varying levels of participation from different stakeholders, but overall, the data-sharing agenda usually is formulated by a handful of stakeholders such as funding agencies and research organizations, in which regional or national regulatory frameworks have been considered on a case-by-case basis. Generally, discussions on biomedical data sharing are conducted from the perspective of those leading the development of the current global infrastructure for health science research, namely, academia, pharmaceutical companies, clinical health IT systems, funders, insurers, and policy-makers. Although all of them would state that they are fundamentally concerned with health and the furtherance of the public good, the vested interests of this infrastructure (i.e., academic achievement, the profit motive, international political competition) may not align with the solutions identified above [24]. This can not only lead to inequalities between researchers in low- and high-resourced countries [25], but also an agenda that does not necessarily embody the concerns of research participants.

To some extent, patient organizations have undertaken similar initiatives to create opportunities for both data generation and sharing. Examples are enabling research participants or patients to directly upload health information or share genetic variants [26], or requiring collaborating investigators to share data as a condition of receiving registry data or biobank samples. While this has become more of the norm in terms of rare diseases, in other areas, this is not necessarily the case. The more commonplace situation for global infrastructure is that the people from whom the data originate, i.e., the research participant, the patient receiving treatment, the family member providing blood, or the community volunteering information, are not routinely provided with the opportunity for involvement in the design and management of data-storage decisions, or sharing practices [27, 28].

## An inclusive and global approach

There is a pressing and growing need for a data-sharing governance framework that is inclusive, global, and transparent. Such a framework should enable the involvement of all of those who have contributed the data and who have an interest in the health research carried out, while still accounting for variations in those interests across different stakeholders. Further, it would stretch beyond national borders, include the lens of responsible data use and accountability, and enable democracy in a pluralistic global community. The key challenge becomes, then, how to reform the national data-sharing governance structure and to scale it up to a global level to produce a structure that is attentive to stakeholders from *all* of the research constituencies (research users, public and participants, industry, policy-makers, and researchers themselves) from *all* of the territories that those stakeholders inhabit (not just the western research paradigm) [29]. Such a perspective will encourage paradigm shifts in the way we manage and disseminate data, even in the face of entrenched systems [30]. To achieve this, we need to orient the discussions about priorities around the individuals from whom the data are obtained and to include a broader range of constituencies in planning and oversight.

Currently, generation and access criteria for data, samples and other materials vary considerably between national and international repositories in terms of permission levels, review bodies and policy requirements [31]. Ensuring global equity in the value of large scale data-sharing will require systems that are used in all countries and recognized by all users and producers of data. The same advances in technology that present the challenges described above can also be part of the solution, enabling a better representation of the various values and cultures of the data providers and the communities they represent. With regards to a technical infrastructure, developing a global governance system that is appropriate for sharing and enabling the appropriate flow of data will require interoperable systems across all countries that are secure, transparent and accountable [32], and accessibility by all contributors, producers, and users of data [32, 33]. While individual systems employed by discrete projects need not be uniform, central or common, they should be harmonized so as to be designed around a transparent set of features that make them compatible with those used elsewhere, and comprehendible to an external audience.

Such systems must enable local and regional and socio-cultural decisions to be made with regard to sharing that respect the participants, and still permit the available data to be shared. Enabled by advances in electronic communication, the basic governance mechanisms that will allow this are in the process of development. Examples are the ORCID identification initiative, building a registry of unique researcher identifiers that could be used as a passport for allowing access to datasets and legitimate uses of data [34]; ‘Dynamic Consent’, an online interface that enables ongoing communication between data volunteers (participants) and users (researchers), and allows consent preferences to be modified over time [35]; and ÉCOUTER, an online tool for engaging and analyzing stakeholder perspectives [36, 37]. Widespread adoption of these systems would promote integrity, accountability, legitimacy, and trust.

Importantly, data-sharing governance structures must include the general public in setting priorities and determining how data are used. If we assume that data sharing is a means to an end, and that end is to benefit all communities equally, then the gold standard of public consultation should be to begin in a place that considers all communities in their own context. This means avoiding hypothetical person-centered approaches that presume a universal ‘reasonable person’ standard: if we want to understand how data sharing should occur in a particular country or region or how research participants can or do contribute to data-sharing planning, we need to do so empirically using a collection of robust and recognized social science approaches [6, 12].

In practice, we have a long way to go to achieving this ambition, but it is a perspective that will provide a strong framework for considering new options and could be transformative in integrating the tensions between the collective and the individual. In some countries, recent initiatives by funders of healthcare research to engage members of their public in decision-making processes have been praised for helping to re-direct research questions towards the needs of patients [38]. It is now widely accepted that research questions and research design benefit from public consultation, and the same approach should apply to the design of data-sharing mechanisms, particularly when drafting data management plans and governance plans for data infrastructure, rather than this being the sole responsibility of researchers. Securing the involvement of both scientific and participant data providers in the decision-making of consortia and data infrastructure is another mechanism to account for publicly funded research activities and ensuring that these voices are heard.

## Conclusion

Good practices for data sharing must evolve towards an interoperable set of standards, permitting sharing across borders. Good practices must also ensure that all views are heard and taken into account when defining these standards. The governance of science will have to become more transparent, representative, and responsive to the voices of many constituencies, with all the prospects for discord, compromise, and delay that this implies. We argue that as research and data-sharing activities are largely publicly funded, there is a strong moral argument for including the people who provide the data in decision-making and to develop governance systems to enable their continued participation. We further argue creative engagement of individual, familial, and community data volunteers with all stakeholders across boundaries, whether disciplinary, institutional, or national, has the potential to generate insights that may otherwise be overlooked or ignored when global perspectives are less comprehensive. This creative engagement can also reduce research waste [39, 40] by removing duplication and avoiding avenues of exploration already known to be dead ends. We also argue that appropriate data sharing can, and does, permit significant advances in our understanding of many complex health-related issues, helping to address currently unmet health needs.

The question is whether we have the political will to develop and facilitate these good data sharing practices. We have the technology that can enable us to do this now, in system examples such as ORCID; ‘Dynamic Consent’; and ÉCOUTER. Funding agencies and research organizations, which recognize the potential efficiency gains from data sharing and are critical in the formulation of the data-sharing agenda should review and revise their data sharing policies to involve participants [41].

When pursued from the perspective of the donors of information, and structured for their benefit, collective exploitation of data between stakeholders and across communities will create the possibility of improving efficiency and more importantly, of addressing inequalities.
